# The Effects of Bioactive Glass-Containing Toothpastes on *Streptococcus mutans* Biofilm Removal from Contemporary Restorative Materials: An In Vitro Study

**DOI:** 10.3390/dj14090597

**Published:** 2026-09-15

**Authors:** Isfendiyar Isfendiyaroglu, Asli Sahiner Daniskan, Cemile Bagkur, Cenk Serhan Ozverel, Mehmet Isfendiyaroglu, Zeynep Ergucu

**Affiliations:** 1 Department of Restorative Dentistry, Faculty of Dentistry, Ege University, 35040 Izmir, Türkiye; isfendiyar.isfendiyaroglu@kstu.edu.tr (I.I.); zergucu@yahoo.com (Z.E.); 2 Department of Biology, Faculty of Science, Ege University, 35040 Izmir, Türkiye; asli.sahiner@ege.edu.tr; 3 DESAM Research Institute, Near East University, TRNC Mersin 10, 99138 Nicosia, Türkiye; cenkserhan.ozverel@neu.edu.tr; 4 Department of Basic Medical Sciences, Faculty of Dentistry, Near East University, TRNC Mersin 10, 99138 Nicosia, Türkiye; 5 Department of Animal Sciences, Faculty of Veterinary Medicine, Near East University, TRNC Mersin 10, 99138 Nicosia, Türkiye; mehmet.isfendiyaroglu@neu.edu.tr

**Keywords:** *Streptococcus mutans*, dental biofilm, toothpaste, bioactive glass, dental restorative material, NovaMin, BioMin F

## Abstract

**Background/Objectives**: Dental biofilms are complex microbial communities that could lead to the formation of dental caries and other oral diseases. This in vitro study aimed to evaluate the effects of two commercially available toothpastes containing bioactive glass (BioMin F and Sensodyne Repair & Protection) on biofilm removal from five contemporary restorative dental materials and bovine enamel. The objective of this study was to compare the effects of bioactive glass-containing toothpastes on *Streptococcus mutans* viability and colonization on different restorative materials and bovine enamel. **Methods**: A total of 42 disc specimens representing five restorative dental materials and bovine enamel were incubated with *Streptococcus mutans* for 24 h to develop biofilms. After bacterial adhesion, specimens were brushed with toothpaste slurries (1:2 dilution) and distilled water (control). Biofilm viability and bacterial colonization were evaluated using the MTT assay and colony-forming unit (CFU) counts, respectively. Biofilm morphology on selected specimens was examined by scanning electron microscopy (SEM). Differences among groups were analyzed statistically using Tukey’s test. **Results**: Statistically significant differences were observed among the study groups following brushing (*p* < 0.05). EQUIA Forte HT (EF) demonstrated the lowest levels of *S. mutans* viability and colonization, whereas ACTIVA PRONTO (AP) exhibited the highest levels. Among the toothbrushing methods, BioMin F resulted in a greater reduction in bacterial viability and colony counts independent of the restorative materials. Combined analysis indicated that EF brushed with Sensodyne Repair & Protect yielded the lowest bacterial burden, whereas AP brushed with distilled water exhibited the highest. The results describing bacterial viability and colony counts also correlate with the SEM images taken. **Conclusions**: Within the limitations of this in vitro study, Sensodyne Repair & Protect was associated with lower *S. mutans* viability and colonization on most restorative materials, particularly EQUIA Forte HT.

## 1. Introduction

Dental biofilm that colonizes tooth surfaces consists of a highly diverse community of microorganisms. Under healthy conditions, this microbial community helps regulate pH balance and prevents colonization by exogenous species. Disruption of this balance can lead to the development of dental caries. Mechanical biofilm control using active agents can positively influence this biological equilibrium [1]. Nanotherapeutics, in which Sensodyne Repair & Protect and BioMin F are the two toothpastes using the same technology, offer a promising approach to control oral biofilm formation [2,3].

The oral cavity is a complex environment harboring over 700 bacterial taxa [4]. A key player in this ecosystem is dental plaque formation, which naturally develops on both hard and soft tissues of the mouth [5,6]. Oral biofilms consist of microbial species embedded in a matrix of bacterial components, salivary proteins/peptides, and food debris. Biofilms are formed by oral bacteria and can cause various localized oral diseases [2,7]. Pathologies such as dental caries or periodontitis may arise when this balance is disrupted, resulting in an imbalance among the resident microorganisms [6].

In recent years, advancements in nanotechnology have generated considerable interest in the development of caries-preventive materials. New bioactive restorative materials combine the advantages of resin-modified glass ionomer cements, conventional glass ionomer cements, and composite resins while mitigating their respective limitations.

Previous studies evaluated remineralization or dentin hypersensitivity, whereas evidence regarding biofilm removal on recently introduced bioactive restorative materials remains scarce. Given the limited data on the interaction between newly developed bioactive glass-containing toothpastes and contemporary bioactive restorative materials [8,9], an in vitro study was designed to evaluate the effects of two toothpastes—containing different bioactive glasses and fluoride—on biofilm removal. The study specifically assessed their impact on bovine enamel, bioactive resin materials, glass ionomer cement, and resin composite surfaces under controlled in vitro conditions.

Therefore, this study aimed to compare the effects of two commercially available bioactive glass-containing toothpastes on *S. mutans* biofilm viability and colonization formed on contemporary restorative materials and bovine enamel using microbiological and ultrastructural analyses. The study’s null hypothesis is that toothpastes containing different bioactive glasses are not more effective than brushing with distilled water in removing biofilm formed on intact bovine enamel surfaces and different restorative materials within 24 h.

## 2. Materials and Methods

### 2.1. Study Design and the Preparation of Enamel and Restorative Material Discs

A total of 252 specimens were prepared across 5 restorative material types (OptiShade, Kerr Dental) (OC), conventional glass ionomer cement (Ketac Molar Easymix, 3M ESPE) (KMG), glass hybrid restorative material (EQUIA Forte HT Fil, GC) (EF), resin-modified glass ionomer cement (ACTIVA BioACTIVE RESTORATIVE, Pulpdent) (AB), and flowable bioactive composite (ACTIVA PRONTO, Pulpdent) (AP) and bovine tooth (42 specimens per product type). From each restorative material type, 3 specimens were initially set aside as untreated baseline controls, with 1 specimen per material allocated for Scanning Electron Microscopy (SEM) surface analysis. The remaining 39 specimens per product type were randomly assigned to 3 different brushing regimens (13 specimens per brushing subgroup (brushing with distilled water, brushing with BioMin F and brushing with Sensodyne Repair) per material type). Within each subgroup (n = 13), samples were allocated to specific analytical assays: n = 6 for the MTT viability assay to evaluate biocompatibility and potential cytotoxic effects, n = 6 for the colony-counting assay (CFU) to quantify bacterial adhesion post-treatment, and n = 1 for qualitative SEM analysis to observe micro-surface topographical alterations. To streamline statistical comparisons, the combination of 6 product types and 3 brushing regimens yielded an 18-level categorical independent variable. The primary quantitative dependent variables were cellular viability and microbial load. The allocation of n = 6 specimens per group for quantitative assays (n = 108 total) was validated through a post hoc power analysis using G*Power (v3.1.9.7); assuming a large effect size (f = 0.40) and an alpha level of α= 0.05, this sample size achieved a statistical power (1 − β) of 85.2%, thereby exceeding the standard 80% threshold.

**Figure 1:**
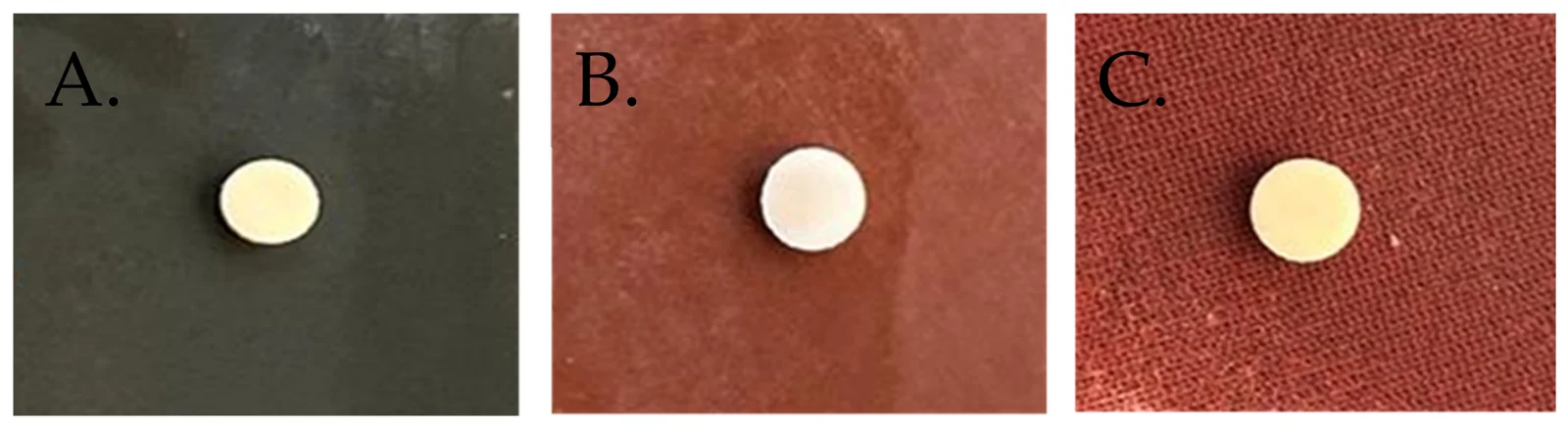
Specimen surfaces were standardized (2 mm thickness, 8 mm diameter) through sequential polishing with 1000- (**A**), 2000- (**B**), and 4000-grit (**C**) SiC abrasive papers.

The surfaces of bovine teeth (BT) obtained from a slaughterhouse were thoroughly cleaned. Enamel discs with a diameter of 8 mm and a thickness of 2 mm were prepared from the enamel surfaces. The teeth were sectioned under continuous water cooling using a high-speed handpiece with diamond burs, and final shaping was performed accordingly (Figure 1). A total of 42 bovine enamel specimens were prepared for the study.

Restorative material samples were prepared as recommended by the manufacturer. Prefabricated plastic molds with inner diameters of 8 mm and 2 mm were used to prepare the specimens. For each restorative material, 42-disc specimens were fabricated, resulting in a total of 210 restorative samples. The type, composition, and manufacturers of the restorative materials used in the study are summarized in Table 1.

### 2.2. Surface Polishing of the Specimens

Restorative material specimens were prepared using plastic molds in accordance with the manufacturers’ instructions. After removal from the molds, the specimens were inspected for air bubbles, and any excess material or flash was removed using a scalpel.

To ensure comparable surface roughness among all groups, specimens were polished using three different silicon carbide abrasive papers. Each specimen was sequentially polished for 30 s with 1000-, 2000-, and 4000-grit abrasive papers under continuous water cooling (Figure 1). Following surface preparation, the specimens were rinsed with distilled water and air-dried using an air–water spray (Figure 1).

### 2.3. Bacterial Adhesion and MTT Assay

*S. mutans* ATCC 25175 was cultured in Brain Heart Infusion (BHI) broth (Merck Millipore, Billerica, MA, USA) at 37 °C for 12 h under 5% CO_2_. Following incubation, the culture was centrifuged at 2200× *g* for 5 min, washed twice with phosphate-buffered saline (PBS), and resuspended. The bacterial suspension was adjusted to a turbidity of 1.0 McFarland using a densitometer.

Specimens were placed into 48-well plates. For one group, 1.5 mL of BHI broth supplemented with 1% sucrose and artificial saliva (1:1) and 100 µL of the bacterial suspension were added, and the samples were incubated under microaerophilic conditions for 24 h to allow for biofilm formation. The saliva used was an artificial saliva (1.5 mM of CaCl_2_, 0.9 mM of KH_2_PO_4_, 130 mM of KCl, 1 mM of NaN_3_, and 20 mmol/L of HEPES) that was adjusted to pH 7 [10].

In the other group, biofilms were formed using 1.5 mL of BHI broth containing 1% sucrose only.

After 24 h of biofilm formation, 1 mL of 3-(4,5-dimethylthiazol-2-yl)-2,5-diphenyltetrazolium bromide (MTT) solution (0.5 mg/mL in PBS) was added to each well and incubated for 1 h. During this period, metabolically active bacteria reduced MTT to formazan crystals, and crystals were dissolved in 500 μL of Dimethyl sulfoxide (DMSO). The plates were incubated in the dark for 20 min, 200 µL aliquots were transferred to 96-well plates, and absorbance was measured at 540 nm [11]. All sample groups were studied as triplicates.

### 2.4. Colony-Forming Unit (CFU) Enumeration

Colony counting was performed to quantitatively determine the viability of microorganisms within the biofilm. Biofilm-coated specimens were transferred into tubes containing 2 mL of PBS, vortexed for 1 min, and subsequently subjected to sonication at 100 W for 15 min to detach adherent bacteria.

Serial dilutions were prepared, and aliquots were plated onto BHI agar using the spread-plate technique. After incubation at 37 °C for 24 h, colonies were counted, and the number of viable bacteria on the specimen surfaces was calculated and expressed as CFU [12].

### 2.5. Brushing Procedure After Bacterial Adhesion

Following biofilm formation, the specimens of each restorative material and bovine enamel were divided into three groups. Each group was brushed for 2 min using one of two toothpastes—BioMin F/Sensodyne Repair & Protect—or distilled water, followed by rinsing with distilled water for 1 min. Toothpaste slurries were prepared by mixing toothpaste and distilled water at a 1:2 ratio in accordance with ISO/TR 14569-1 guidelines for dental wear testing. The slurry was continuously delivered to the specimen surface to mimic intraoral salivary dilution and abrasive action [1]. Toothpaste compositions are provided in Table 2.

The brushing procedure was performed using a rechargeable electric toothbrush (Oral-B GENIUS 9000; Procter & Gamble, Cincinnati, OH, USA) fixed onto a custom flat platform to ensure standardized brushing geometry and eliminate operator variability. The toothbrush was operated in the “Pro Clean” mode for all experimental groups, delivering approximately 8800 oscillations and 40,000 pulsations per minute. To maintain consistency across treatments, Oral-B CrossAction brush heads were employed, and a new brush head was utilized for each distinct experimental group. A constant downward load of 200 g (1.96 N) was continuously applied to the specimens using a calibrated vertical loading mechanism, representing the clinically recommended average manual/oscillating brushing force to prevent severe hard tissue or restoration wear.

### 2.6. An Evaluation of the Effect of Brushing on Biofilm Removal

To quantitatively assess the viability of microorganisms within the biofilm on brushed specimens, colony counting was performed. After the brushing procedure, the specimens were rinsed with sterile distilled water and transferred into tubes containing 2 mL of PBS. The samples were vortexed for 1 min and subsequently subjected to sonication at 100 W for 15 min to detach adherent bacteria.

Serial dilutions were then prepared, and aliquots were plated onto BHI agar using the spread-plate method. Following incubation at 37 °C for 24 h, colonies were counted, and the number of viable bacteria present on the specimen surfaces was calculated and expressed as CFU [12].

### 2.7. Scanning Electron Microscopy (SEM) Analysis of Specimen Surfaces

For SEM analysis, the prepared disc specimens were allocated to groups for brushing after bacterial adhesion. From each set of 39 specimens, three samples were selected for SEM analysis for brushing with distilled water, brushing with Biomin F, brushing with Sensodyne Repair, along with one specimen from an untreated group, which no brushing is applied to (n = 4 for each restorative material).

Following bacterial adhesion and brushing procedures, the prepared discs were sputter-coated with gold using a QUORUM Q150 RES coating system and examined using a Carl Zeiss 300 VP scanning electron microscope (Carl Zeiss, Jena, Germany) at various magnifications to evaluate bacterial adhesion on the specimen surfaces.

### 2.8. Statistical Analysis

The results of the microbiological and biological analyses were statistically evaluated using the NCSS 2007 Statistical Software (NCSS LLC, Kaysville, UT, USA). Descriptive statistics, including the mean and standard deviation [Mean ± SD], were calculated for all variables. Prior to inferential analysis, data distribution was assessed for normality using the Shapiro–Wilk test alongside visual inspection of Q-Q plots, and the homogeneity of variances across experimental groups was verified using Levene’s test. Upon confirming that the parametric test assumptions of normality and homoscedasticity were satisfied, intergroup comparisons were conducted using one-way analysis of variance (ANOVA). Tukey’s HSD multiple comparison test was subsequently applied for post hoc analysis to identify specific subgroup differences. All statistical evaluations were conducted at a significance level of *p* < 0.05.

## 3. Results

### 3.1. MTT Results of S. mutans According to Material Type and Brushing Method Following Bacterial Adhesion

**Figure 2:**
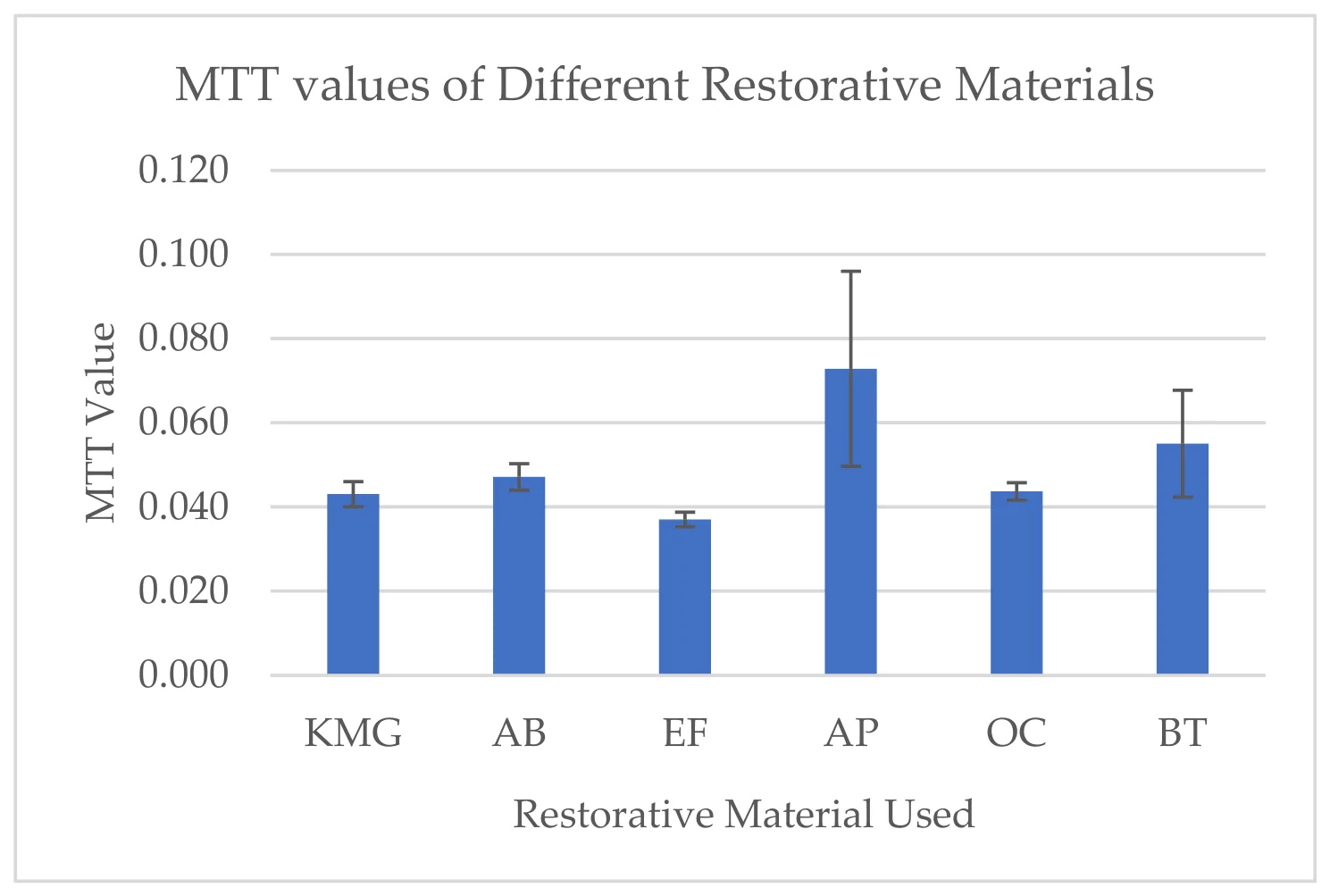
Comparison of *S. mutans* MTT assay values across different restorative materials. (OC: Nanohybrid universal composite, KMG: conventional glass ionomer cement, EF: glass hybrid restorative material, AB: resin-modified glass ionomer cement, AP: flowable bioactive composite, BT: bovine tooth).

**Figure 3:**
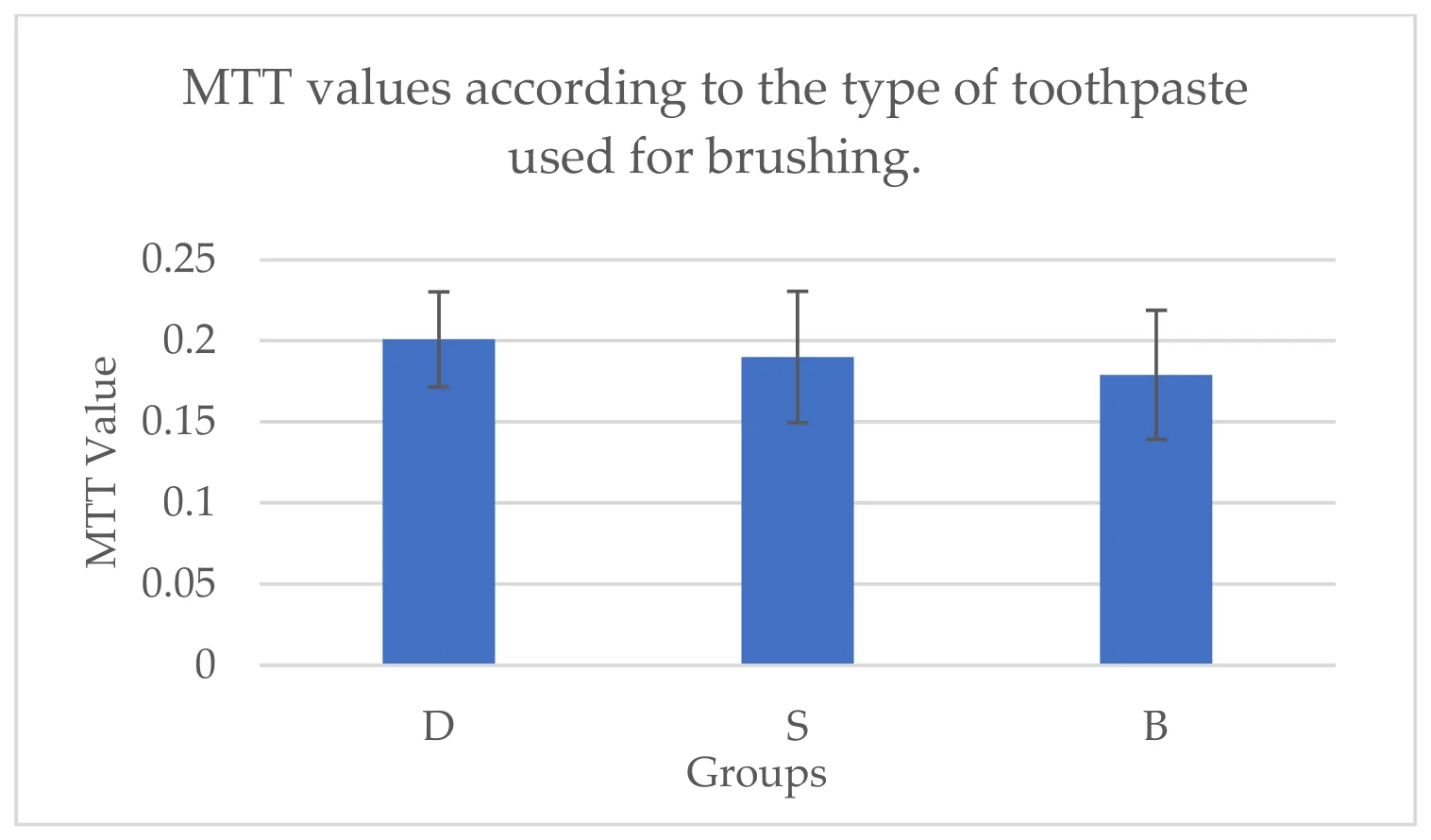
Comparison of *S. mutans* MTT values according to the type of toothpaste used for brushing. (D: distilled water, S: Sensodyne Repair & Protect, B: BioMin F).

MTT values were analyzed to determine differences among restorative materials irrespective of brushing method and among brushing methods regardless of material type. ANOVA followed by Tukey’s HSD test revealed statistically significant differences in bacterial viability among different groups. Among the materials, the highest bacterial viability was observed in the AP group, while the lowest was detected in the EF group (Figure 2). Bacterial viability varied according to brushing method, and brushing with distilled water showed the highest bacterial viability, whereas brushing with BioMin F demonstrated the lowest viability value (Figure 3).

Significant differences in bacterial adhesion were observed among restorative materials (Table 3; Additional results are provided in Supplementary Table S1). The AP group demonstrated the significantly highest adhesion of *S. mutants (p* < 0.001), followed by the BT group, which also demonstrated higher adhesion of *S. mutants* in comparison to all other test groups, except the AP group *(p* < 0.001), whereas the EF group showed the significantly lowest adhesion (*p* < 0.001).

Regarding brushing methods (Table 4; Additional results are provided in Supplementary Table S2), samples brushed using the B method (brushing with BioMin F) exhibited significantly lower bacterial adhesion compared to the D (brushing with distilled water) and S methods (brushing with Sensodyne Repair), while no significant difference was observed between the D and S methods.

Compared with control samples, all groups demonstrated reduced bacterial viability following brushing after bacterial adhesion (Table 5).

### 3.2. Effect of Restorative Material Type and Brushing Method on S. mutans Colony Numbers

Colony numbers of *S. mutans* within the biofilm formed on restorative materials were analyzed to determine whether significant differences existed according to material type, irrespective of brushing method, and according to brushing method regardless of material type. ANOVA followed by Tukey’s HSD post hoc test revealed statistically significant differences in colony formation among the groups (*p* < 0.05) (Table 6).

**Figure 4:**
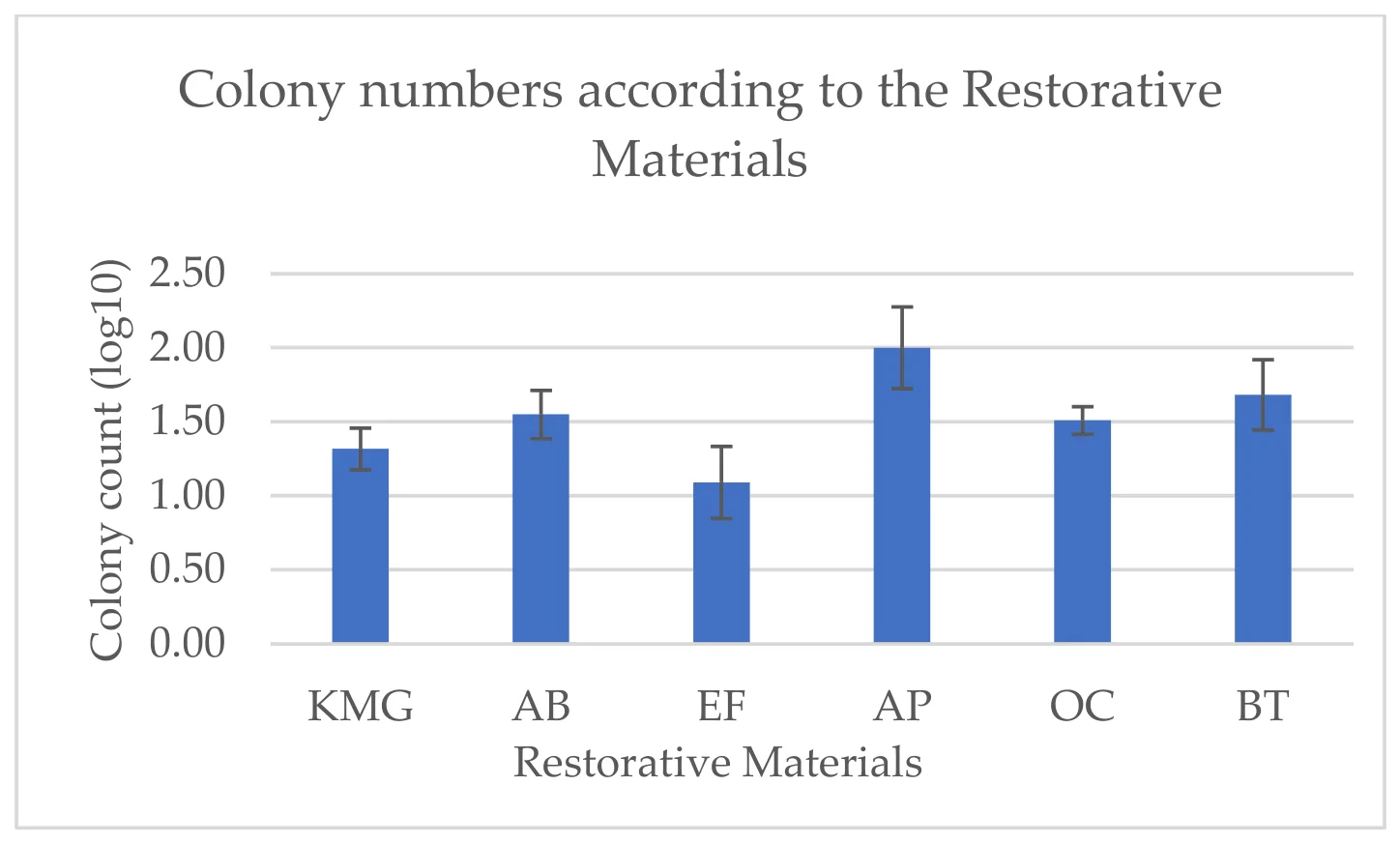
Comparison of *S. mutans* colony-count values across different restorative materials. (OC: Nanohybrid universal composite, KMG: conventional glass ionomer cement, EF: glass hybrid restorative material, AB: resin-modified glass ionomer cement, AP: flowable bioactive composite, BT: bovine tooth).

**Figure 5:**
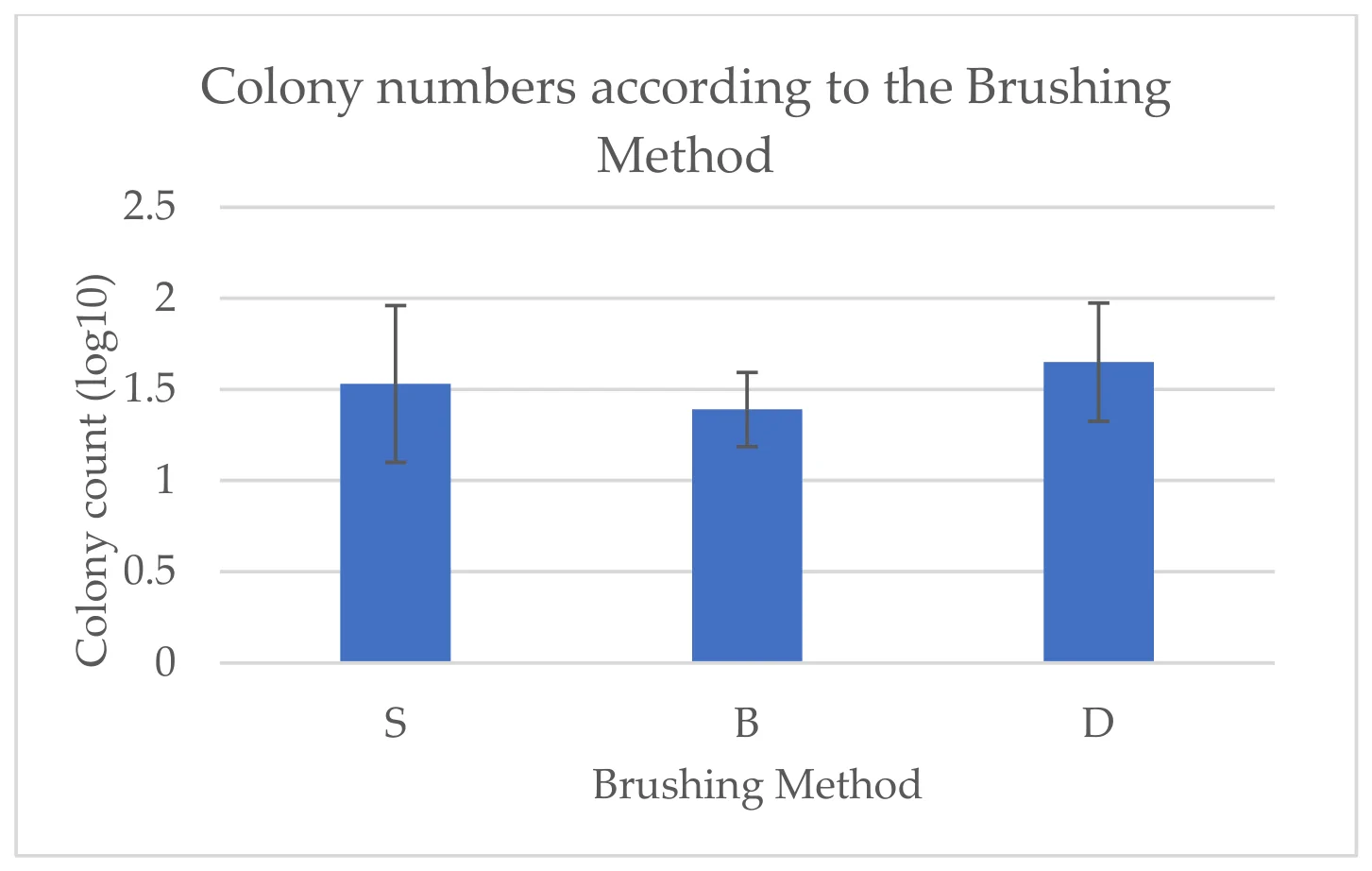
Comparison of *S. mutans* colony count values between different brushing methods. (D: distilled water, S: Sensodyne Repair & Protect, B: BioMin F).

Among the restorative materials, the AP group exhibited the highest colony formation, whereas the EF group showed the lowest colony counts (Figure 4). Among the brushing methods, the D method resulted in the highest colony formation, whereas the B method produced the lowest colony counts (Figure 5).

Pairwise comparisons indicated significant differences among all restorative materials (Table 7; Additional results are provided in Supplementary Table S3). The AP group showed the significantly greatest colony formation (*p* < 0.001), while the EF group significantly exhibited the lowest levels of bacterial colony numbers (*p* < 0.001). Significant differences were also observed among brushing methods (Table 8; Additional results are provided in Supplementary Table S4), with the D method resulting in higher colony counts compared to both B and S methods (*p* < 0.001).

Compared with control samples, all groups showed reduced colony counts following brushing after bacterial adhesion, indicating the effectiveness of brushing in decreasing *S. mutans* biofilm formation (Table 9).

### 3.3. MTT Results of S. mutans According to the Combination of Material Type and Brushing Method Following Bacterial Adhesion

**Figure 6:**
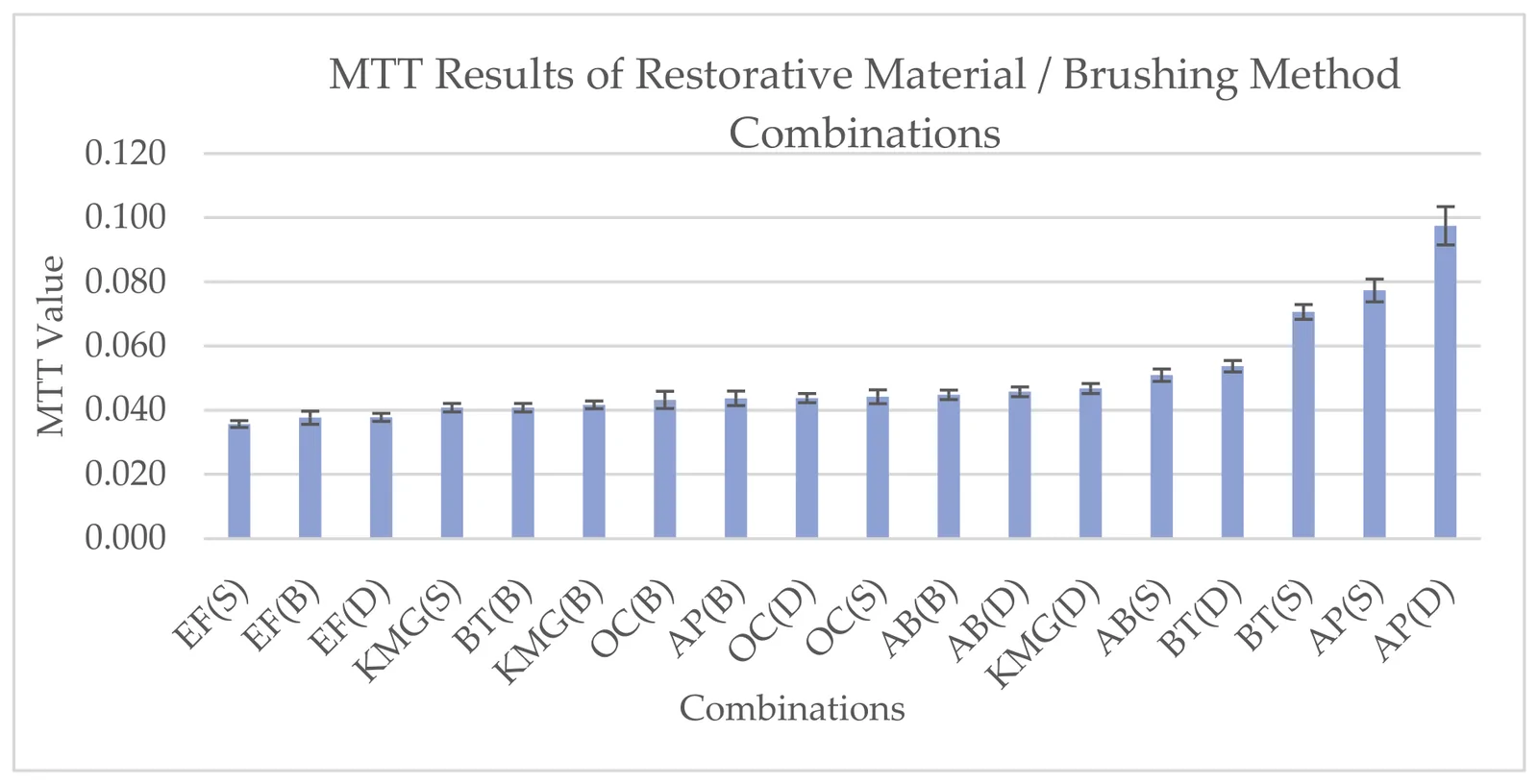
Comparison of MTT values of *S. mutans* that remain attached on different restorative materials (groups) and that were subjected to one of the 3 different brushing methods post-bacterial adhesion. (OC(D): Nanohybrid universal composite with distilled water, OC(B): Nanohybrid universal composite with BioMin F, OC(S): Nanohybrid universal composite with Sensodyne Repair; KMG(D): Conventional glass ionomer cement with distilled water, KMG(B): Conventional glass ionomer cement with BioMin F, KMG(S): Conventional glass ionomer cement with Sensodyne Repair; EF(D): glass hybrid restorative material with distilled water, EF(B): glass hybrid restorative material with BioMin F, EF (S): glass hybrid restorative material with Sensodyne Repair, AB(D): Resin-modified glass ionomer cement with distilled water, AB(S): Resin-modified glass ionomer cement with Sensodyne Repair, AB(B): Resin-modified glass ionomer cement with BioMin F; AP(D): Flowable bioactive composite with distilled water, AP(S): Flowable bioactive composite with Sensodyne Repair, AP(B): Flowable bioactive composite with BioMin F; BT(D): Bovine tooth with distilled water, BT(S): Bovine tooth with Sensodyne Repair, BT(B): Bovine tooth with BioMin F).

Differences in *S. mutans* MTT values among groups formed by combinations of restorative materials and brushing methods were statistically evaluated. Mean MTT values of the six parallels for each material–brushing combination were calculated, and statistically significant differences among groups were identified. The highest bacterial viability was observed in the AP(D) group, whereas the lowest viability was detected in the EF(S) group, which demonstrated the most effective combination of restorative material and brushing method (Figure 6). Brushing AP restorative material with Sensodyne Repair significantly reduced bacterial viability in comparison to brushing of the same material with BioMin F (*p* < 0.05) (Table 10).

Overall, EF-based groups exhibited significantly lower MTT values compared with most other material–brushing combinations, indicating reduced bacterial adhesion (Figure 6). In contrast, combinations involving AP, particularly AP(D), consistently demonstrated higher MTT values, reflecting increased bacterial viability. Intermediate levels of viability were observed among KMG, AB, BT, and OC groups, depending on the brushing method applied (Table 10).

### 3.4. Colony-Count Results of S. mutans According to Material–Brushing Method Combinations Following Bacterial Adhesion

**Figure 7:**
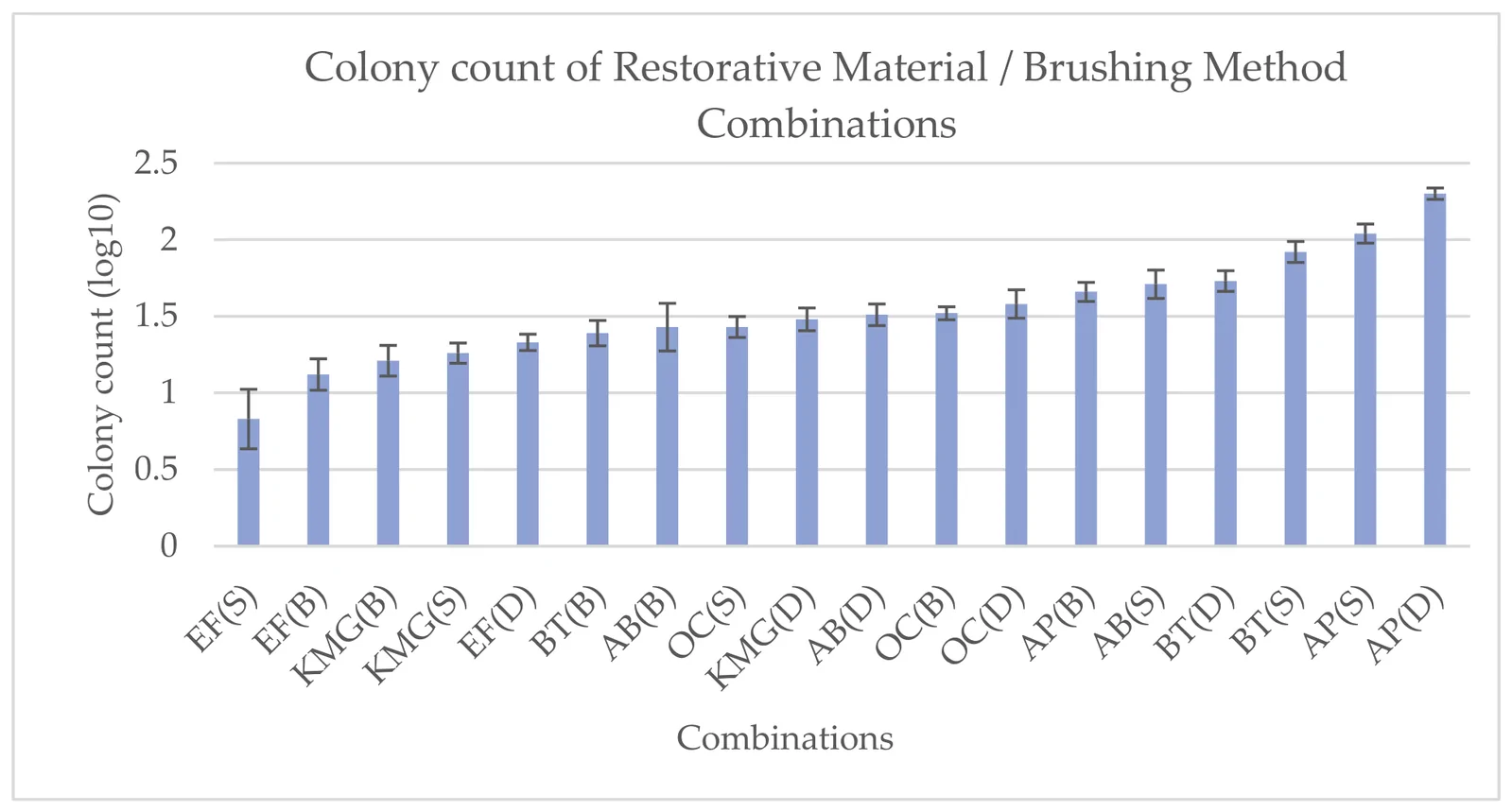
Comparison of colony count (log10) of *S. mutans* on different restorative materials (groups) that were subjected to one of the 3 different brushing methods post-bacterial adhesion. (OC(D): Nanohybrid universal composite with distilled water, OC(B): Nanohybrid universal composite with BioMin F, OC(S): Nanohybrid universal composite with Sensodyne Repair; KMG(D): Conventional glass ionomer cement with distilled water, KMG(B): Conventional glass ionomer cement with BioMin F, KMG(S): Conventional glass ionomer cement with Sensodyne Repair; EF(D): glass hybrid restorative material with distilled water, EF(B): glass hybrid restorative material with BioMin F, EF (S): glass hybrid restorative material with Sensodyne Repair, AB(D): Resin-modified glass ionomer cement with distilled water, AB(S): Resin-modified glass ionomer cement with Sensodyne Repair, AB(B): Resin-modified glass ionomer cement with BioMin F; AP(D): Flowable bioactive composite with distilled water, AP(S): Flowable bioactive composite with Sensodyne Repair, AP(B): Flowable bioactive composite with BioMin F; BT(D): Bovine tooth with distilled water, BT(S): Bovine tooth with Sensodyne Repair, BT(B): Bovine tooth with BioMin F).

The number of *S. mutans* colonies formed within the biofilm on restorative materials was evaluated to determine whether there were statistically significant differences among groups created by the combinations of restorative materials and brushing methods (Table 11). The highest mean colony count was observed in the AP(D) group, whereas the lowest was detected in the EF(S) group (Figure 7). Post hoc analysis identified multiple statistically significant differences between material–brushing combinations. Overall, EF(S) exhibited significantly lower colony counts compared with all other groups (*p* < 0.05), while AP(D) showed significantly higher colony counts than all test groups (*p* < 0.05).

### 3.5. SEM Analysis of S. mutans Following Brushing After Bacterial Adhesion

**Figure 8:**
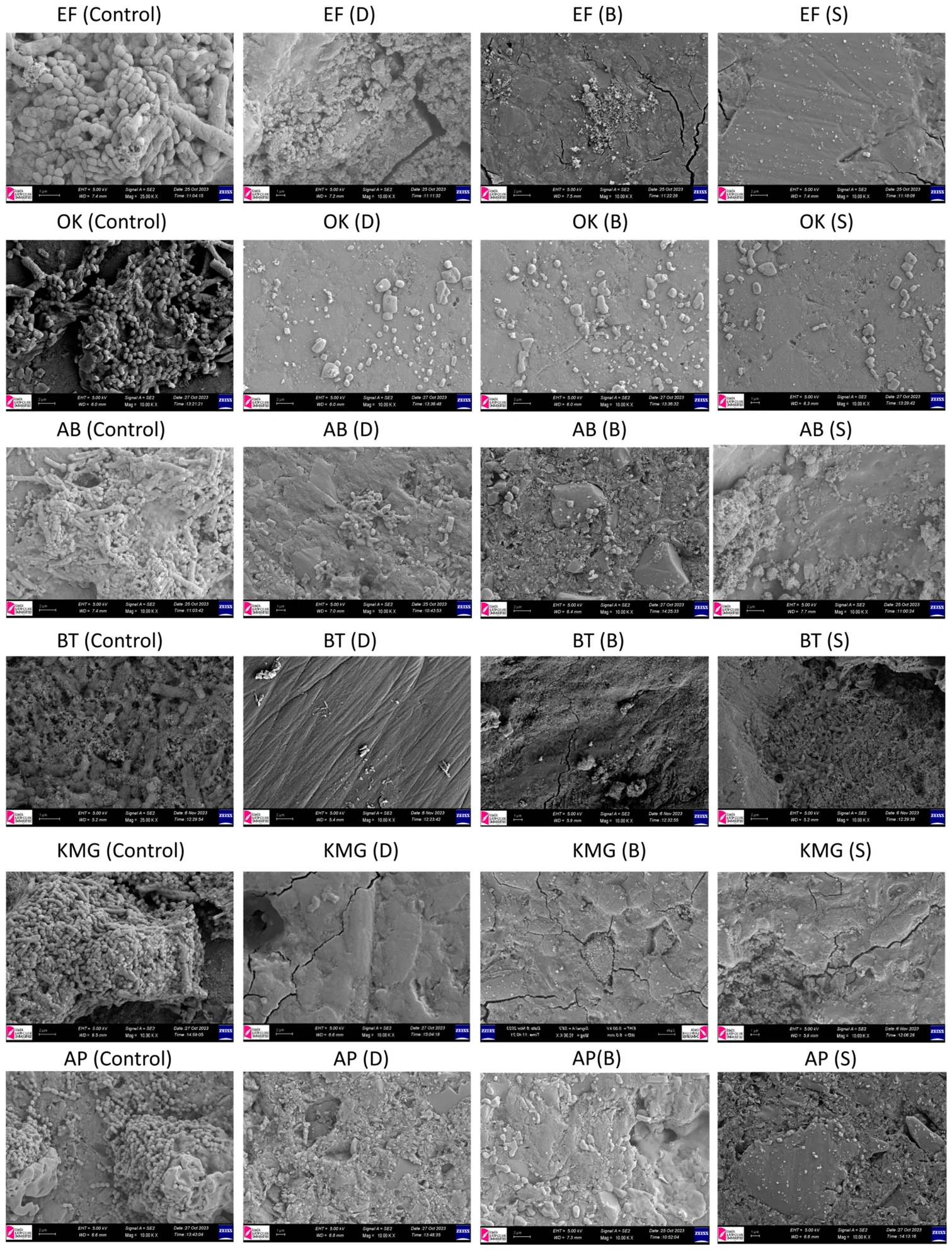
SEM images of discs of restorative material or extracted bovine tooth after 24 h *S. mutans* colonization (control groups) and images of discs of restorative material or extracted bovine tooth after 24 h *S. mutans* colonization, followed by 2 min of different brushing methods (×10,000 magnification). (Control groups: glass hybrid restorative material control (EF(control)), Nanohybrid universal composite control (OC(control)), Resin-modified glass ionomer cement control (AB(control)), BT(control), Conventional glass ionomer cement control (KMG (control)), Flowable bioactive composite control (AP(control)); OC(D): Nanohybrid universal composite with distilled water, OC(B): Nanohybrid universal composite with BioMin F, OC(S): Nanohybrid universal composite with Sensodyne Repair; KMG(D): Conventional glass ionomer cement with distilled water, KMG(B): Conventional glass ionomer cement with BioMin F, KMG(S): Conventional glass ionomer cement with Sensodyne Repair; EF(D): glass hybrid restorative material with distilled water, EF(B): glass hybrid restorative material with BioMin F, EF (S): glass hybrid restorative material with Sensodyne Repair, AB(D): Resin-modified glass ionomer cement with distilled water, AB(S): Resin-modified glass ionomer cement with Sensodyne Repair, AB(B): Resin-modified glass ionomer cement with BioMin F; AP(D): Flowable bioactive composite with distilled water, AP(S): Flowable bioactive composite with Sensodyne Repair, AP(B): Flowable bioactive composite with BioMin F; BT(D): Bovine tooth with distilled water, BT(S): Bovine tooth with Sensodyne Repair, BT(B): Bovine tooth with BioMin F).

*S. mutans* colonization activity was further assessed by participating a SEM analysis on post-incubated discs after applying various brushing methods. The image analysis clearly demonstrated that all the study groups, including the bovine-tooth group, demonstrated a lower colonization of *S. mutans* after the application of one of the brushing methods, including brushing with distilled water. EF restorative material has demonstrated the lowest *S. mutans* colonization, especially when brushed with Sensodyne Repair & Protect, demonstrating a correlation with MTT and colony-count analyses (Figure 6 and Figure 7). SEM images also demonstrated that the lowest colonization after brushing was reported for the BT study group, independent of brushing method. When restorative material groups were investigated independently, brushing with Sensodyne Repair & Protect demonstrated the lowest level of colonization (Figure 8).

## 4. Discussion

In the present study, the effects of bioactive glass-containing toothpastes on *S. mutans* viability and colonization on different restorative materials and bovine enamel were studied. *S. mutans* was selected due to its strong adhesion capability to dental and restorative surfaces [13,14] and its pronounced acidogenic and aciduric properties, which make it one of the most cariogenic oral microorganisms [13,15].

Bacterial biofilms are three-dimensional structures embedded in an extracellular polysaccharide matrix, and biofilm accumulation on restorative materials not only increases surface roughness but also facilitates bacterial colonization at restoration margins [16,17]. This may accelerate lesion progression and increase the frequency of restoration replacement. Therefore, there is growing interest in restorative materials and oral care products that reduce bacterial adhesion or release antimicrobial and remineralizing ions [18,19].

Mechanical plaque control (groups of samples that are brushed with a toothbrush only) alone has been reported to be insufficient for complete biofilm removal; therefore, chemical plaque control using toothpaste is considered an essential adjunct [20,21]. Fluoride is the most important active ingredient in toothpaste due to its remineralizing effects and its ability to inhibit bacterial metabolism [22,23]. Fluoride promotes the conversion of hydroxyapatite into the more acid-resistant fluorapatite, thereby increasing enamel resistance to acidic challenges [24,25]. Additionally, fluoride may alter plaque biofilm composition and reduce cariogenicity [26]. However, concerns regarding the long-term use of fluoride and other antimicrobial agents have increased interest in alternative bioactive formulations [27].

Conventional bioactive glass formulations are limited in fluoride content [28]. Recently developed fluoride-containing bioactive glass toothpastes have been reported to provide sustained release of calcium, phosphate, and fluoride ions, enhancing remineralization and potentially reducing bacterial adhesion [29]. Although several studies have evaluated the effects of different toothpastes on bacterial adhesion [30,31], data regarding bioactive glass-containing toothpastes remain scarce.

The aim of our study is to determine the effectiveness of current bioactive glass-based toothpastes (Sensodyne Repair & Protect and BioMin F) in reducing *S. mutans* in an in vitro environment and to understand how beneficial it might be to brush different restorative materials with these toothpastes. In this way, patients in the clinic can be given advice on which toothpaste will be the better option for them depending on the restorative material they have.

The results indicate that there are material-related differences in bacterial behavior independent of toothpaste application. In this regard, AP was reported to exhibit the highest levels of viable *S. mutans* adhesion and colony formation. The AP group demonstrated significantly higher *S. mutans* colony-number values in comparison to all other study groups. In contrast, the lowest levels of viable bacterial adhesion and colonization were observed in the EF glass hybrid restorative material followed by the KMG group [32]. The toothpastes included in this study contain fluoride, which inhibits bacterial acid production by suppressing glycolytic enzymes, thereby increasing plaque pH. In addition, fluoride has a high affinity for calcium, which interferes with the adhesion of phosphates and proteins to hydroxyapatite, ultimately reducing bacterial adhesion and colonization [33,34]. The low levels of viable bacterial adhesion and colonization observed in the EF and KMG groups after brushing following bacterial adhesion might be associated with their glass ionomer-based structure, fluoride content, and their ability to be recharged with fluoride from toothpaste during brushing [35].

When brushing methods were compared, regardless of the sample being brushed, the lowest colony count and live bacterial adhesion were observed with brushing using BioMin F toothpaste. This might be associated with the integration of fluoride ions alongside calcium and phosphate within BioMin F’s bioactive glass structure to form fluorhydroxyapatite, which enables a sustained delivery of fluoride, particularly in a neutral pH environment [36]. Fluoride is widely recognized for its anti-cariogenic efficacy, effectively targeting cariogenic pathogens like *S. mutans* [37]. It disrupts the bacterial cell membrane and functions as a metabolic inhibitor; as protons subsequently acidify the cytoplasm, bacterial cell death occurs [38]. Furthermore, an in vitro study demonstrated that the calcium- and phosphate-based crystalline matrix inherently inhibits *S. mutans* biofilm formation [39]. Additionally, according to the manufacturer, the fluorhydroxyapatite formed by BioMin F exhibits a ten-fold greater resistance to acid dissolution compared to the hydroxyapatite generated by NovaMin [40].

An assessment of the toothpaste–material combinations revealed differences in bacterial colonization patterns, with EF demonstrating the lowest levels of *S. mutans* colonization and viable bacterial adhesion. The lowest values were observed in the EF group brushed with Sensodyne Repair & Protect, EF(S). In contrast, the AP group brushed with distilled water, AP(D), demonstrated the highest levels of both viable bacterial adhesion and colonization and was statistically significantly different from all other groups.

Both toothpastes used in this study contained bioactive glass; however, Sensodyne Repair & Protect has a higher fluoride content and different bioactive glass composition, particle size and morphology, dentifrice abrasiveness, and formulation pH than BioMin F, which might explain the differences in the results. The significantly lower colonization and viable bacterial adhesion observed in the EF(S) group, including lower values compared with BioMin F-brushed specimens, may therefore be attributed to the aforementioned differences in the Sensodyne Repair & Protect group [33]. Another reason for low colonization adhesion in the EF(S) group might be due to the fact that each toothpaste has a different paste matrix, and it is known that BioMin F contains fluoride as fluorocalcium phosphosilicate with sustained release of ingredients [41].

In the evaluation of viable bacterial adhesion and colonization, the EF groups were followed by the conventional glass ionomer cement KMG groups brushed with both toothpastes. No statistically significant difference was observed between the EF groups and the KMG(S) and KMG(B) groups. Sensodyne could provide not only remineralization but also a direct antibacterial effect. BioMin F, on the other hand, focuses more on remineralization through controlled calcium/phosphate/fluoride release, and its antibacterial effect is more indirect and limited. Therefore, Sensodyne might be more advantageous in biofilm suppression. The relatively low bacterial adhesion observed in the KMG groups may also be related to their fluoride-containing composition.

Consistent with our findings, Albannawi (2016) reported that conventional glass ionomer cements exhibit higher antimicrobial activity than ACTIVA BioACTIVE-RESTORATIVE, while ACTIVA materials show antimicrobial properties comparable to those of compomers and composite resins [42]. In the present study, materials with more pronounced glass ionomer characteristics demonstrated lower bacterial adhesion after brushing following bacterial adhesion.

The higher colonization and viable bacterial adhesion observed in the AP(D) group may partly be related to the brushing protocol; however, the similarly high values observed in the AP(S) group suggest that factors beyond brushing method contribute to this outcome. Increased colonization after post-adhesion brushing indicates a stronger affinity of *S. mutans* for the AP surface. As no previous studies have evaluated biofilm formation on AP, further interpretation regarding its surface characteristics remains limited. AP(B) also shows high colonization and viable bacterial adhesion; this could be explained by its resin matrix. Although the material exhibits favorable bioactive properties, the presence of a substantial organic resin phase can compromise its long-term surface integrity. Resin-based matrices are prone to water sorption and subsequent hydrolytic degradation, which eventually leads to the formation of micro voids and increased surface heterogeneity [43,44]. Furthermore, these hydrophobic/hydrophilic irregularities accelerate non-specific protein adsorption [45], establishing a precursor pellicle layer that facilitates bacterial retention within the newly formed micro voids [46].

Following the AP groups, bovine enamel brushed with Sensodyne Repair & Protect, BT(S), and distilled water, BT(D), showed the next highest colonization numbers. A statistically significant difference was observed between BT(S) and BT(D), with higher viable bacterial adhesion in the BT(S) group. This might be attributed to the alteration of surface topography and increased surface roughness (Ra) induced by the deposition of bioactive glass particles (Calcium Sodium Phosphosilicate/NovaMin) present in the BT(S) formulation [47,48]. According to the established threshold in the dental literature, an increase in surface roughness exceeding 0.2 μm physically facilitates bacterial entrapment and initial plaque retention [49]. Furthermore, the rapid dissolution of bioactive glass in aqueous environments releases a high concentration of divalent calcium ions into the micro-environment. These cations act as ionic bridges that neutralize the electrostatic repulsion between the negatively charged bovine enamel and the bacterial cell walls, thereby accelerating viable bacterial adhesion in the BT(S) group compared to the ion-free distilled-water control [50].

AP’s bioactive structure is known to exhibit hydroxyapatite-like ionic interactions. The enamel is also hydroxyapatite in structure. Therefore *S. mutans* might be using similar adhesion mechanisms on both surfaces. Bacteria might have a better ability to bind to surfaces with medium-to-high surface energy [51]. The enamel and AP might be behaving in a similar surface energy range. This might explain the similar colonization results on both surfaces.

The effects of recently developed toothpaste formulations with bioactive glasses on biofilm formations were also assessed in the study, and the results highlight their role in influencing bacterial colonization and biofilm dynamics [3]. In addition to their mechanical cleaning action, toothpastes serve two primary functions: acting as antimicrobial adjuncts to facilitate the removal of pathogenic dental biofilm and forming a protective barrier on tooth surfaces to enhance resistance against future dental and periodontal diseases [52,53].

Although several studies have investigated dentin hypersensitivity outcomes associated with Sensodyne Repair & Protect and BioMin F toothpastes, data regarding their effects on bacterial adhesion are lacking. In the present study, the effectiveness of these two bioactive glass-containing toothpastes in preventing *S. mutans* adhesion and removing established biofilm on different restorative materials was evaluated.

Previous studies assessing the effects of various toothpastes on *S. mutans* have consistently demonstrated that fluoride-containing formulations exhibit antibacterial activity and reduce bacterial adhesion [30,31,54,55]. Our findings contribute novel data by extending this evidence to bioactive glass-based toothpaste formulations.

In the present study, the effects of bioactive glass-containing toothpastes on *S. mutans* viability and colonization on different restorative materials and bovine enamel were studied; however, further investigation of the bioactive materials tested in this study is needed, as their precise compositions remain incompletely defined, limiting a full understanding of their bioactive mechanisms. Surface-roughness and ion-releasing-capacity measurements were not performed, and even though the oral cavity is polymicrobial, only *S. mutans* were used as target microorganisms in this study, and both are accepted as limitations of this study.

## 5. Conclusions

Among the tested materials, EF glass hybrid restorative material combined with Sensodyne Repair & Protect, EF(S), showed the most effective synergy for biofilm removal, while AP flowable bioactive composite with Sensodyne, AP(S), was the least effective combination after AP(D). Therefore, the results reject the null hypothesis suggested. However, these findings are based on an in vitro study, and they do not reflect clinical outcomes as they do not fully reflect the oral environment. Limitations of the present study include the use of only an *S. mutans* biofilm model and the absence of an acquired pellicle, oral pH changes, aging of materials tested, surface-roughness and ion-release evaluations.

Within the limitations of this study, Sensodyne Repair & Protect was associated with lower *S. mutans* viability and colonization on the majority of restorative materials tested, particularly EQUIA Forte HT. Future studies should explore the long-term clinical effects of these toothpastes on biofilm control and their interaction with different restorative materials in vivo.

## Figures and Tables

**Table 1:** Types, compositions, and manufacturers of the restorative materials used in this study.

Materials	Type	Composition	Manufacturers
OptiShade (OC)	Nanohybrid Composite Resin	BisGMA, BisEMA, TEGDMA, Zirconia, and silica nanoparticles.	Kerr, Orange, CA, USA
Ketac Molar (KMG)	Traditional Glass Ionomer	Liquid: Polyalkenoic acid, tartaric acid, and water Powder: Fluoroaluminosilicate glass and Al–Ca–La copolymer.	3 M ESPE, St. Paul, MN, USA
EQUIA Forte HT Fil (EF)	Glass Hybrid Restorative Material	Liquid: Polyacrylic acid, distilled water, and polybasic carboxylic acid Powder: Strontium fluoroaluminosilicate glass, polyacrylic acid powder, and pigment.	GC Corp., TOCio, Tokyo, Japan
ACTIVA BioACTIVE-RESTORATIVE (AB)	Resin-Modified Glass Ionomer	Diurethane modified by the addition of hydrogenated polybutadiene and other methacrylate monomers, polyacrylic acid, silica, and sodium fluoride.	Pulpdent, Watertown, MA, USA
ACTIVA PRONTO (AP)	Flowable Bioactive Composite	A mixture of diurethane and other methacrylate resins; silica, amorphous.	Pulpdent, Watertown, MA, USA

**Table 2:** Toothpastes used in the study, including their manufacturers, compositions, and active ingredients.

Toothpaste	Manufacturer	Composition	Active Ingredient
Sensodyne Repair & Protect (S)	Glaxo Smith Kline, Brentford, Middlesex, UK	Glycerin, PEG-8, aqueous silica, calcium sodium phosphosilicate (NovaMin), pentasodium triphosphate, sodium lauryl sulfate, sweetener, titanium dioxide, polyacrylic acid, cocamidopropyl betaine, sodium saccharin, and sodium fluoride 0.3152% *w*/*w* (1450 ppm).	NovaMin
BioMin F (B)	BioMin Technologies Ltd., London, UK	Fluoro calcium silicate, sodium lauryl sulfate, titanium dioxide, acesulfame potassium, carbomer, polyethylene glycol 400 (PEG 400), glycerin, silica, flavor, and fluoride < 600 ppm.	BioMin F (fluoro calcium phosphosilicate) fluoride content: <600 ppm F

**Table 3:** Groups showing differences in *S. mutans* MTT values among the tested materials (*p* < 0.05). (OC: Nanohybrid universal composite, KMG: conventional glass ionomer cement, EF: glass hybrid restorative material, AB: resin-modified glass ionomer cement, AP: flowable bioactive composite, BT: bovine tooth).

Restorative Material	KMG	AB	EF	AP	OC	BT
*p* < 0.05						
KMG		<0.001	<0.001	<0.001		<0.001
AB	<0.001		<0.001	<0.001	<0.001	<0.001
EF	<0.001	<0.001		<0.001	<0.001	<0.001
AP	<0.001	<0.001	<0.001		<0.001	<0.001
OC		<0.001	<0.001	<0.001		<0.001
BT	<0.001	<0.001	<0.001	<0.001	<0.001	

**Table 4:** Groups showing differences in *S. mutans* MTT values according to brushing methods (*p* < 0.05) (D: distilled water, S: Sensodyne Repair & Protect, B: BioMin F).

MTT Brushing	D	S	B
*p* < 0.05			
D			<0.001
S			<0.001
B	<0.001	<0.001	

**Table 5:** *S. mutans* MTT values of discs subjected to brushing after bacterial adhesion, mean MTT values of materials independent of brushing method (Mean A), and mean MTT values of bacteria according to brushing method independent of material (Mean B). (OC: Nanohybrid universal composite, KMG: conventional glass ionomer cement, EF: glass hybrid restorative material, AB: resin-modified glass ionomer cement, AP: flowable bioactive composite, BT: bovine tooth).

MTT	Brushing Method																			
Restorative Material	S						B						D						Mean A	Control Groups
KMG	0.041	0.042	0.040	0.041	0.043	0.039	0.042	0.042	0.041	0.042	0.043	0.040	0.047	0.048	0.046	0.047	0.049	0.045	0.043	0.261
AB	0.051	0.053	0.049	0.050	0.054	0.048	0.045	0.046	0.044	0.045	0.047	0.043	0.046	0.047	0.045	0.046	0.048	0.044	0.047	0.305
EF	0.036	0.036	0.035	0.036	0.037	0.034	0.038	0.034	0.037	0.039	0.037	0.040	0.038	0.039	0.037	0.038	0.040	0.036	0.037	0.346
AP	0.077	0.082	0.075	0.076	0.081	0.073	0.044	0.045	0.043	0.045	0.046	0.040	0.097	0.092	0.104	0.106	0.094	0.092	0.073	0.120
OC	0.044	0.045	0.041	0.042	0.046	0.047	0.043	0.044	0.046	0.046	0.039	0.041	0.044	0.045	0.043	0.044	0.046	0.042	0.044	0.229
BT	0.071	0.072	0.069	0.070	0.074	0.067	0.041	0.042	0.040	0.041	0.043	0.039	0.054	0.055	0.052	0.053	0.056	0.051	0.055	0.155
Mean B	0.053						0.042						0.054							

**Table 6:** *S. mutans* colony counts on discs subjected to brushing after bacterial adhesion, mean colony counts on materials independent of brushing method (Mean _a_), and mean colony counts of bacteria according to brushing method independent of material (Mean _b_). (OC: Nanohybrid universal composite, KMG: conventional glass ionomer cement, EF: glass hybrid restorative material, AB: resin-modified glass ionomer cement, AP: flowable bioactive composite, BT: bovine tooth).

Colony Numbers	Brushing Method																			
Restorative Material	S						B						D						Mean A	Control Groups
KMG	1.15	1.30	1.32	1.26	1.30	1.23	1.08	1.34	1.26	1.18	1.30	1.14	1.46	1.54	1.34	1.54	1.46	1.50	1.32	8
AB	1.76	1.68	1.87	1.65	1.71	1.61	1.59	1.34	1.64	1.31	1.40	1.27	1.56	1.49	1.53	1.46	1.61	1.42	1.55	8.18
EF	0.90	0.70	0.95	0.66	1.11	0.63	0.95	1.20	1.03	1.17	1.21	1.13	1.28	1.34	1.34	1.31	1.41	1.27	1.09	8.28
AP	2.01	2.08	1.96	2.05	2.14	2.01	1.63	1.69	1.59	1.65	1.76	1.62	2.27	2.32	2.32	2.29	2.35	2.25	2.00	7.61
OC	1.36	1.48	1.35	1.44	1.53	1.40	1.51	1.57	1.55	1.53	1.45	1.50	1.61	1.56	1.57	1.52	1.75	1.48	1.51	7.96
BT	1.89	1.96	1.85	1.92	2.02	1.89	1.28	1.43	1.34	1.40	1.52	1.36	1.79	1.71	1.72	1.67	1.84	1.67	1.68	7.68
Mean B	1.53						1.39						1.65							

**Table 7:** Groups showing statistically significant differences in *S. mutans* colony counts among the materials (OC: Nanohybrid universal composite, KMG: conventional glass ionomer cement, EF: glass hybrid restorative material, AB: resin-modified glass ionomer cement, AP: flowable bioactive composite, BT: bovine tooth).

Colony Counts on Restorative Materials	KMG	AB	EF	AP	OC	BT
*p* < 0.05						
KMG		<0.001	<0.001	<0.001	<0.001	<0.001
AB	<0.001		<0.001	<0.001		<0.001
EF	<0.001	<0.001		<0.001	<0.001	<0.001
AP	<0.001	<0.001	<0.001		<0.001	<0.001
OC	<0.001		<0.001	<0.001		<0.001
BT	<0.001	<0.001	<0.001	<0.001	<0.001	

**Table 8:** Groups showing statistically significant differences in *S. mutans* colony counts according to brushing methods (D: distilled water, S: Sensodyne Repair & Protect, B: BioMin F).

Colony Counts Across Different Brushing Methods	D	S	B
*p* < 0.05			
D		<0.001	<0.001
S	<0.001		<0.001
B	<0.001	<0.001	

**Table 9:** *S. mutans* colony counts on discs subjected to brushing after bacterial adhesion, mean colony counts on materials independent of brushing method (Mean A), and mean colony counts according to brushing method independent of material (Mean B). (OC: Nanohybrid universal composite, KMG: conventional glass ionomer cement, EF: glass hybrid restorative material, AB: resin-modified glass ionomer cement, AP: flowable bioactive composite, BT: bovine tooth).

Colony Count	Brushing Method																			
Restorative Materials	S						B						D						Mean A	Control Groups
KMG	1.15	1.30	1.32	1.26	1.30	1.23	1.08	1.34	1.26	1.18	1.30	1.14	1.46	1.54	1.34	1.54	1.46	1.50	1.32	8
AB	1.76	1.68	1.87	1.65	1.71	1.61	1.59	1.34	1.64	1.31	1.40	1.27	1.56	1.49	1.53	1.46	1.61	1.42	1.55	8.18
EF	0.90	0.70	0.95	0.66	1.11	0.63	0.95	1.20	1.03	1.17	1.21	1.13	1.28	1.34	1.34	1.31	1.41	1.27	1.09	8.28
AP	2.01	2.08	1.96	2.05	2.14	2.01	1.63	1.69	1.59	1.65	1.76	1.62	2.27	2.32	2.32	2.29	2.35	2.25	2.00	7.61
OC	1.36	1.48	1.35	1.44	1.53	1.40	1.51	1.57	1.55	1.53	1.45	1.50	1.61	1.56	1.57	1.52	1.75	1.48	1.51	7.96
BT	1.89	1.96	1.85	1.92	2.02	1.89	1.28	1.43	1.34	1.40	1.52	1.36	1.79	1.71	1.72	1.67	1.84	1.67	1.68	7.68
Mean B	1.53						1.39						1.65							

**Table 10:** In samples where brushing was performed after bacterial adhesion, the mean MTT values and statistically significant differences were reported in the groups (*p* < 0.05). ns: not significant (OC(D): Nanohybrid universal composite with distilled water, OC(B): Nanohybrid universal composite with BioMin F, OC(S): Nanohybrid universal composite with Sensodyne Repair; KMG(D): Conventional glass ionomer cement with distilled water, KMG(B): Conventional glass ionomer cement with BioMin F, KMG(S): Conventional glass ionomer cement with Sensodyne Repair; EF(D): glass hybrid restorative material with distilled water, EF(B): glass hybrid restorative material with BioMin F, EF (S): glass hybrid restorative material with Sensodyne Repair, AB(D): Resin-modified glass ionomer cement with distilled water, AB(S): Resin-modified glass ionomer cement with Sensodyne Repair, AB(B): Resin-modified glass ionomer cement with BioMin F; AP(D): Flowable bioactive composite with distilled water, AP(S): Flowable bioactive composite with Sensodyne Repair, AP(B): Flowable bioactive composite with BioMin F; BT(D): Bovine tooth with distilled water, BT(S): Bovine tooth with Sensodyne Repair, BT(B): Bovine tooth with BioMin F).

MTT Value and Significant Differences, Sig. *p* < 0.05																		
Control	0.346	0.346	0.346	0.261	0.155	0.261	0.229	0.120	0.229	0.229	0.305	0.305	0.261	0.305	0.155	0.155	0.120	0.120
Average	0.036	0.038	0.038	0.041	0.041	0.042	0.043	0.044	0.044	0.044	0.045	0.046	0.047	0.051	0.054	0.071	0.077	0.097
Material/Brushing	EF(S)	EF(B)	EF(D)	KMG(S)	BT(B)	KMG(B)	OC(B)	AP(B)	OC(D)	OC(S)	AB(B)	AB(D)	KMG(D)	AB(S)	BT(D)	BT(S)	AP(S)	AP(D)
EF(S)																		
EF(B)	ns																	
EF(D)	ns	ns																
KMG(S)	ns	ns	ns															
BT(B)	ns	ns	ns	ns														
KMG(B)	0.004	ns	ns	ns	ns													
OC(B)	<0.001	ns	ns	ns	ns	ns												
AP(B)	<0.001	0.002	ns	ns	ns	ns	ns											
OC(D)	<0.001	0.001	0.004	ns	ns	ns	ns	ns										
OC(S)	<0.001	0.001	0.003	ns	ns	ns	ns	ns	ns									
AB(B)	<0.001	<0.001	<0.001	ns	ns	ns	ns	ns	ns	ns								
AB(D)	<0.001	<0.001	<0.001	ns	ns	ns	ns	ns	ns	ns	ns							
KMG(D)	<0.001	<0.001	<0.001	0.004	0.004	ns	ns	ns	ns	ns	ns	ns						
AB(S)	<0.001	<0.001	<0.001	<0.001	<0.001	<0.001	<0.001	<0.001	<0.001	0.001	ns	ns	ns					
BT(D)	<0.001	<0.001	<0.001	<0.001	<0.001	<0.001	<0.001	<0.001	<0.001	<0.001	<0.001	<0.001	0.001	ns				
BT(S)	<0.001	<0.001	<0.001	<0.001	<0.001	<0.001	<0.001	<0.001	<0.001	<0.001	<0.001	<0.001	<0.001	<0.001	<0.001			
AP(S)	<0.001	<0.001	<0.001	<0.001	<0.001	<0.001	<0.001	<0.001	<0.001	<0.001	<0.001	<0.001	<0.001	<0.001	<0.001	<0.001		
AP(D)	<0.001	<0.001	<0.001	<0.001	<0.001	<0.001	<0.001	<0.001	<0.001	<0.001	<0.001	<0.001	<0.001	<0.001	<0.001	<0.001	<0.001	

**Table 11:** In samples brushed after bacterial adhesion, mean colony counts and statistically significant differences were reported in the groups (*p* < 0.05). ns: not significant. (OC(D): Nanohybrid universal composite with distilled water, OC(B): Nanohybrid universal composite with BioMin F, OC(S): Nanohybrid universal composite with Sensodyne Repair; KMG(D): Conventional glass ionomer cement with distilled water, KMG(B): Conventional glass ionomer cement with BioMin F, KMG(S): Conventional glass ionomer cement with Sensodyne Repair; EF(D): glass hybrid restorative material with distilled water, EF(B): glass hybrid restorative material with BioMin F, EF (S): glass hybrid restorative material with Sensodyne Repair, AB(D): Resin-modified glass ionomer cement with distilled water, AB(S): Resin-modified glass ionomer cement with Sensodyne Repair, AB(B): Resin-modified glass ionomer cement with BioMin F; AP(D): Flowable bioactive composite with distilled water, AP(S): Flowable bioactive composite with Sensodyne Repair, AP(B): Flowable bioactive composite with BioMin F; BT(D): Bovine tooth with distilled water, BT(S): Bovine tooth with Sensodyne Repair, BT(B): Bovine tooth with BioMin F).

Colony Value and Significant Differences, Sig. *p* < 0.05																		
Control	8.28	8.28	8.00	8.00	8.28	7.68	8.18	7.96	8.00	8.18	7.96	7.96	7.61	8.18	7.68	7.68	7.61	7.61
Average	0.83	1.12	1.21	1.26	1.33	1.39	1.43	1.43	1.48	1.51	1.52	1.58	1.66	1.71	1.73	1.92	2.04	2.3
Material/Brushing	EF(S)	EF(B)	KMG(B)	KMG(S)	EF(D)	BT(B)	AB(B)	OC(S)	KMG(D)	AB(D)	OC(B)	OC(D)	AP(B)	AB(S)	BT(D)	BT(S)	AP(S)	AP(D)
EF(S)																		
EF(B)	<0.001																	
KMG(B)	<0.001	ns																
KMG(S)	<0.001	ns	ns															
EF(D)	<0.001	ns	ns	ns														
BT(B)	<0.001	<0.001	ns	ns	ns													
AB(B)	<0.001	<0.001	ns	ns	ns	ns												
OC(S)	<0.001	<0.001	ns	ns	ns	ns	ns											
KMG(D)	<0.001	<0.001	<0.001	ns	ns	ns	ns	ns										
AB(D)	<0.001	<0.001	<0.001	0.001	ns	ns	ns	ns	ns									
OC(B)	<0.001	<0.001	<0.001	<0.001	ns	ns	ns	ns	ns	ns								
OC(D)	<0.001	<0.001	<0.001	<0.001	<0.001	ns	ns	ns	ns	ns	ns							
AP(B)	<0.001	<0.001	<0.001	<0.001	<0.001	<0.001	0.003	0.003	ns	ns	ns	ns						
AB(S)	<0.001	<0.001	<0.001	<0.001	<0.001	<0.001	<0.001	<0.001	0.002	ns	ns	ns	ns					
BT(D)	<0.001	<0.001	<0.001	<0.001	<0.001	<0.001	<0.001	<0.001	<0.001	ns	ns	ns	ns	ns				
BT(S)	<0.001	<0.001	<0.001	<0.001	<0.001	<0.001	<0.001	<0.001	<0.001	<0.001	<0.001	<0.001	<0.001	ns	ns			
AP(S)	<0.001	<0.001	<0.001	<0.001	<0.001	<0.001	<0.001	<0.001	<0.001	<0.001	<0.001	<0.001	<0.001	<0.001	<0.001	ns		
AP(D)	<0.001	<0.001	<0.001	<0.001	<0.001	<0.001	<0.001	<0.001	<0.001	<0.001	<0.001	<0.001	<0.001	<0.001	<0.001	<0.001	<0.001	

## Data Availability

The data obtained from this study are presented in the article and Supplementary Materials. Further inquiries can be directed to the corresponding author.

## Supplementary Materials

The following supporting information can be downloaded at: https://www.mdpi.com/article/10.3390/dj14090597/s1, Table S1: Groups showing differences in S. mutans average MTT values with standard errors among the tested materials; Table S2: Groups showing differences in average S. mutans MTT values with standard errors according to brushing method; Table S3: Groups showing statistically significant differences in average S. mutans colony counts with standard error values among the materials; Table S4: Groups showing average differences in S. mutans colony counts including standard error values according to brushing methods.
