# Supplementary material for: The Effects of Bioactive Glass-Containing Toothpastes on Streptococcus mutans Biofilm Removal from Contemporary Restorative Materials: An In Vitro Study
Source: Dent J (Basel). 2026 Sep 15;14(9):597. doi: 10.3390/dj14090597 (PMC13605020; doi:10.3390/dj14090597)
Supplement: Supplementary file 1 [file dentistry-14-00597-s001.zip › dentistry-4429092-supplementary.pdf]

Table S1. Groups showing differences in *S. mutans* average MTT values with standard errors among the tested materials. (OC: Nanohybrid universal composite, KMG: conventional glass ionomer cement, EF: glass hybrid restorative material, AB: resin-modified glass ionomer cement, AP: flowable bioactive composite, BT: bovine tooth).

Multiple Comparisons						
Dependent variable: MTT 2						
Tukey HSD						
(I) Material	(J) Material	Average difference(I-J)	Std. Error	Sig.	%95 Confidence Interval	
					Lower limit	Upper limit
KMG	AB	-.00406*	.000796	.000	-.00637	-.00174
	BT	-.01178*	.000796	.000	-.01410	-.00946
	EF	.00617*	.000796	.000	.00385	.00848
	OC	-.00056	.000796	.982	-.00287	.00176
	AP	-.02967*	.000796	.000	-.03198	-.02735
AB	KMG	.00406*	.000796	.000	.00174	.00637
	BT	-.00772*	.000796	.000	-.01004	-.00540
	EF	.01022*	.000796	.000	.00790	.01254
	OC	.00350*	.000796	.000	.00118	.00582
	AP	-.02561*	.000796	.000	-.02793	-.02329
BT	KMG	.01178*	.000796	.000	.00946	.01410

	AB	.00772*	.000796	.000	.00540	.01004
	EF	.01794*	.000796	.000	.01563	.02026
	OC	.01122*	.000796	.000	.00890	.01354
	AP	-.01789*	.000796	.000	-.02021	-.01557
EF	KMG	-.00617*	.000796	.000	-.00848	-.00385
	AB	-.01022*	.000796	.000	-.01254	-.00790
	BT	-.01794*	.000796	.000	-.02026	-.01563
	OC	-.00672*	.000796	.000	-.00904	-.00440
	AP	-.03583*	.000796	.000	-.03815	-.03352
OC	KMG	.00056	.000796	.982	-.00176	.00287
	AB	-.00350*	.000796	.000	-.00582	-.00118
	BT	-.01122*	.000796	.000	-.01354	-.00890
	EF	.00672*	.000796	.000	.00440	.00904
	AP	-.02911*	.000796	.000	-.03143	-.02679
AP	KMG	.02967*	.000796	.000	.02735	.03198
	AB	.02561*	.000796	.000	.02329	.02793
	BT	.01789*	.000796	.000	.01557	.02021
	EF	.03583*	.000796	.000	.03352	.03815

	OC	.02911*	.000796	.000	.02679	.03143
Based on observed averages.<br>Error term Mean Squared (Error) = .000						
*. The average difference is statistically significant at the 0.05 level.						

Table S2. Groups showing differences in average *S. mutans* MTT values with standard errors according to brushing method. (D: distilled water, S: Sensodyne Repair & Protect, B: BioMin F).

Multiple Comparisons						
Dependent variable: MTT 2						
Tukey HSD						
(I) Brushing method	(J) Brushing method	Average difference(I-J)	Std. Error	Sig.	%95 Confidence Interval	
					Lower limit	Upper limit
S	B	.01122*	.000563	.000	.00988	.01256
	D	-.00108	.000563	.138	-.00242	.00026
B	S	-.01122*	.000563	.000	-.01256	-.00988
	D	-.01231*	.000563	.000	-.01365	-.01096

D	S	.00108	.000563	.138	-.00026	.00242
	B	.01231*	.000563	.000	.01096	.01365
Based on observed averages.<br>Error term Mean Squared (Error) = .000.						
*. The average difference is statistically significant at the 0.05 level.						

Table S3. Groups showing statistically significant differences in average S. mutans colony counts with standard error values among the materials (OC: Nanohybrid universal composite, KMG: conventional glass ionomer cement, EF: glass hybrid restorative material, AB: resin-modified glass ionomer cement, AP: flowable bioactive composite, BT: bovine tooth).

Multiple Comparisons						
Dependent variable: COLONY COUNT 2						
Tukey HSD						
(I) Material	(J) Material	Average difference(I-J)	Std. Error	Sig.	%95 Confidence Interval	
					Lower limit	Upper limit
KMG	AB	-.2333*	.03004	.000	-.3208	-.1459
	BT	-.3644*	.03004	.000	-.4519	-.2770
	EF	.2283*	.03004	.000	.1409	.3158
	OC	-.1922*	.03004	.000	-.2797	-.1047

	AP	-.6828*	.03004	.000	-.7703	-.5953
AB	KMG	.2333*	.03004	.000	.1459	.3208
	BT	-.1311*	.03004	.000	-.2186	-.0436
	EF	.4617*	.03004	.000	.3742	.5491
	OC	.0411	.03004	.745	-.0464	.1286
	AP	-.4494*	.03004	.000	-.5369	-.3620
BT	KMG	.3644*	.03004	.000	.2770	.4519
	AB	.1311*	.03004	.000	.0436	.2186
	EF	.5928*	.03004	.000	.5053	.6803
	OC	.1722*	.03004	.000	.0847	.2597
	AP	-.3183*	.03004	.000	-.4058	-.2309
EF	KMG	-.2283*	.03004	.000	-.3158	-.1409
	AB	-.4617*	.03004	.000	-.5491	-.3742
	BT	-.5928*	.03004	.000	-.6803	-.5053
	OC	-.4206*	.03004	.000	-.5080	-.3331
	AP	-.9111*	.03004	.000	-.9986	-.8236
OC	KMG	.1922*	.03004	.000	.1047	.2797
	AB	-.0411	.03004	.745	-.1286	.0464

	BT	-.1722*	.03004	.000	-.2597	-.0847
	EF	.4206*	.03004	.000	.3331	.5080
	AP	-.4906*	.03004	.000	-.5780	-.4031
AP	KMG	.6828*	.03004	.000	.5953	.7703
	AB	.4494*	.03004	.000	.3620	.5369
	BT	.3183*	.03004	.000	.2309	.4058
	EF	.9111*	.03004	.000	.8236	.9986
	OC	.4906*	.03004	.000	.4031	.5780
Based on observed averages. Error term Mean Squared (Error) = .008.						
*. The average difference is statistically significant at the 0.05 level.						

Table S4. Groups showing average differences in S. mutans colony counts including standard error values according to brushing methods (D: distilled water, S: Sensodyne Repair & Protect, B: BioMin F).

Multiple Comparisons
Dependent variable: COLONY COUNT 2
Tukey HSD

(I) Brushing method	(J) Brushing method	Average difference(I-J)	Std. Error	Sig.	%95 Confidence Interval	
					Lower limit	Upper limit
S	B	.1447*	.02124	.000	.0941	.1953
	D	-.1228*	.02124	.000	-.1734	-.0722
B	S	-.1447*	.02124	.000	-.1953	-.0941
	D	-.2675*	.02124	.000	-.3181	-.2169
D	S	.1228*	.02124	.000	.0722	.1734
	B	.2675*	.02124	.000	.2169	.3181

Based on observed averages.

Error term Mean Squared (Error) = .008.

*. The average difference is statistically significant at the 0.05 level.
